# Molecular Characterization of the Liver-Expressed Antimicrobial Peptide 2 (LEAP2) from *Amphiprion ocellaris* and Its Role in Antibacterial Immunity

**DOI:** 10.3390/ani15172590

**Published:** 2025-09-03

**Authors:** Dapeng Yu, Tao Li, Kang Wang, Meiling Zhang, Jingyi Mo, Jianlin Chen, Hongli Xia, Yishan Lu, Jia Cai

**Affiliations:** 1 Guangdong Provincial Engineering Research Center for Aquatic Animal Health Assessment, Shenzhen Public Service Platform for Evaluation of Marine Economic Animal Seedings, Shenzhen Institute of Guangdong Ocean University, Shenzhen 518108, China; yudapeng@gdou.edu.cn (D.Y.); 19859722591@163.com (M.Z.); 3105708138@163.com (J.M.); jianlin-chen@m.scnu.edu.cn (J.C.); xiahongli0427@163.com (H.X.); 2 Guangdong Provincial Key Laboratory of Aquatic Animal Disease Control and Healthy Culture, College of Fisheries, Guangdong Ocean University, Zhanjiang 518108, China; 3 Shenzhen Base of South China Sea Fisheries Research Institute, Chinese Academy of Fishery Sciences, Shenzhen 518121, China; lit151415@126.com; 4 Jiangsu Lianshen Ocean Technology, Co., Ltd., Lianyungang 222000, China; 343056369@163.com

**Keywords:** LEAP2, *Amphiprion ocellaris*, antibacterial activity, bacterial infection

## Abstract

Liver-expressed antimicrobial peptide 2 (LEAP2) is a cationic peptide that is integral to the innate immune system. However, its regulatory function in the immunity of fish against pathogens remains largely unexplored. This study identifies a LEAP2 homolog (AoLEAP2) in clownfish, *Amphiprion ocellaris*. Our phylogenetic analysis places AoLEAP2 within the fish LEAP2 cluster, closely related to *Tachinotus anak* LEAP2. AoLEAP2 mRNA is present across various tissues, with the highest expression in the spleen. Following Vibrio harveyi infection, the AoLEAP2 mRNA levels significantly increased in the liver, spleen, gills, and intestine. The chemically synthesized AoLEAP2 mature peptide exhibits broad-spectrum antibacterial activity without displaying toxicity to FHM cells in vitro. The intraperitoneal injection of AoLEAP2 significantly suppresses the mRNA expression of the pro-inflammatory factors IL1β and TNFα induced by *V. harveyi* infection, demonstrating its anti-inflammatory and immune-regulatory role.

## Simple Summary

Diseases caused by harmful bacteria are a serious problem in fish farming, often leading to major losses and requiring antibiotics, which can create other issues like drug resistance. This study looked at a natural defense molecule called LEAP2 found in clownfish, a popular ornamental fish. We wanted to understand how this molecule helps the fish fight infections and if it could be used to protect them better. We discovered that LEAP2 is present in many parts of the clownfish, especially in organs important for fighting disease. When the fish were infected with a common harmful bacteria called *Vibrio harveyi*, the levels of LEAP2 increased significantly in key areas like the liver, spleen, gills, and gut. We made the active part of the LEAP2 molecule in the lab and found it could kill many different types of bacteria without harming the fish’s own cells. Giving this molecule to infected clownfish reduced harmful swelling in their bodies and helped more fish survive the infection. This shows that LEAP2 is a crucial part of the clownfish’s natural defenses. Understanding how it works could lead to new, safer ways to treat diseases in farmed fish, reducing the need for antibiotics and helping protect valuable species like clownfish.

## 1. Introduction

The first antimicrobial peptides (AMPs), also known as host defense peptides (HDPs), were isolated from *Bacillus* spp. soil samples in 1939 [1]. These peptides act as a primary defense mechanism against pathogens and are crucial to the innate immune system [2]. Research has shown that AMPs have a variety of biological functions, including antibacterial activity against both Gram-positive and Gram-negative bacteria; antiviral, antifungal, and antiparasitic effects; tumor cell destruction; and immunoregulation [3]. AMPs are categorized into four secondary structure types—α-helical, β-stranded, β-hairpin or loop, and extended forms [4]—of which α-helical and β-stranded structures are most common. Recent studies have indicated that AMPs can significantly reduce antibiotic overuse in aquaculture, improve the quality of aquatic products, and support sustainable aquaculture development [5], positioning them as promising antibiotic alternatives.

Liver-expressed antimicrobial peptide (LEAP) is a cationic, cysteine-rich peptide initially identified in mammals [6]. Two variants, LEAP1 and LEAP2, have been isolated from human blood ultra-filtrate [7]. LEAP1 contains four disulfide bonds, while LEAP2 has two, and both are predominantly expressed in the liver. These peptides exhibit broad-spectrum antibacterial activity against Gram-positive and Gram-negative bacteria [8]. LEAP2 was first identified in *Oncorhynchus mykiss* (rainbow trout), where two types exist, LEAP2A and LEAP2B, indicating diversity among fish species. This suggests that LEAP2 exhibits substantial variation across different species of fish. Subsequently, LEAP2 genes have been identified in various fish, including *Megalobrama amblycephala*, *Larimichthys crocea*, *Paralichthys olivaceus*, *Ictalurus punctatus*, and *Ctenopharyngodon idellus* [9,10,11,12]. In particular, extensive research has been conducted on LEAP2 in *M. amblycephala* and *P. olivaceus*. The amino acid sequence alignment of *M. amblycephala* reveals that LEAP2 shares the highest similarity with grass carp (95%) and zebrafish (84%) and the lowest similarity with amphibians (*Xenopus tropicalis*, 43%) and mammals (*Homo sapiens*, 38%; *Mus musculus*, 42%) [13]. Systematic evolutionary analysis shows that the LEAP2 gene in *P. olivaceus* exhibits 61.7% homology with *Arothron nigropunctatus*, but only 40.1% with zebrafish. The phylogenetic tree of LEAP2 in fish indicates that they cluster into a distinct branch, reflecting high evolutionary homology with fish and low homology with birds and mammals. This suggests that the LEAP2 gene remained highly conserved in fish during their evolution [13].

Cysteine residues have long been recognized as a critical structural feature of antimicrobial peptides, playing a pivotal role in their antimicrobial function through the formation of disulfide bonds, similar to the role of LEAP1/hepcidin in LEAP2 [14,15]. However, recent evidence suggests that the antimicrobial activity of LEAP2 is not solely dependent on its disulfide bond structure [16]. While disulfide bonds contribute to protein stability, other factors have also been reported to influence LEAP2’s antimicrobial effects. Interestingly, the mature peptide sequence of LEAP2 has remained highly conserved throughout the evolutionary process from lower to higher animals, including the disulfide bond formed by four cysteine residues [17]. Additionally, a high concentration of LEAP2 is required to exert antimicrobial effects, typically much lower than the physiological levels found in blood, suggesting that LEAP2 may have other conserved biological functions beyond its antimicrobial role.

The expression of LEAP2 in fish is influenced by bacterial stimuli. Following injection of the pathogen *Aeromonas hydrophila*, LEAP2 mRNA levels significantly increased in the liver, spleen, brain, and gills of the Chinese bighead carp [18]. Similarly, bacterial challenge led to elevated LEAP2 transcription in the intestine, spleen, and liver of rainbow trout and grass carp [11,18]. The cationic nature and disulfide bonds of LEAP2 enable it to form pores in the membranes of certain Gram-positive and Gram-negative bacteria, exerting a bactericidal effect [19]. The synthetic human LEAP-2-(38–77) polypeptide significantly inhibited the growth of *Bacillus subtilis*, *Bacillus megatherium*, and *Saccharomyces cerevisiae*, but had no effect on *Escherichia coli* and *Pseudomonas fluorescens*, indicating selective antibacterial activity in vitro [20]. This selective antibacterial property is also exhibited by the recombinant LEAP2 expression in *M. amblycephala* [9]. Interestingly, the truncated human LEAP2 (44–77) lacks antibacterial activity, suggesting that the amino-terminal region is crucial for its in vitro antimicrobial function [9]. Alanine scanning mutations further identified Thr2, Phe4, Trp5, and Arg6 as key residues mediating LEAP2’s interactions [21]. The C-terminal sequence of LEAP2 appears to be unrelated to its antibacterial activity but may stabilize the protein in plasma by forming two disulfide bonds (Cys54-Cys65 and Cys60-Cys70) [22]. In vitro, LEAP2 primarily exerts bactericidal effects by disrupting cell membrane integrity [19] or directly penetrating the cell membrane to target genomic DNA for hydrolysis [23]. Under physiological conditions, LEAP2 regulates immune responses through various mechanisms to confer broad-spectrum antibacterial activity characterized by rapid action [24]. For instance, injecting LEAP2 protein into mudflat fish (*Boleophthalmus pecinirostris*) can reduce bacterial load and enhance the antibacterial activity of mononuclear cells/macrophages (MO/MΦ) by inducing chemotaxis, enhancing respiratory bursts, and inhibiting the expression of pro-inflammatory factors [24,25].

Clownfish (*A*. *ocellaris*) are notable habitants of sea anemones with considerable commercial value as ornamental fish, and they are extensively bred in aquaria [26]. However, disease outbreaks involving bacteria (*Vibrio* sp., *Aliivibrio* sp., *Bacillus* sp.), viruses (*Alloherpesvirus*, *Lymphocystis*, *Singapore group iridovirus*), and parasites (*Flagellates*, *Monogeneans*, *Amyloodinium*, *Cryptocaryon*) pose significant challenges to clownfish farming [27]. Recent studies have highlighted the potent antibacterial and antiviral properties of antimicrobial peptides, with LEAP2 recognized as an effective bacteriostatic agent in various fish species. Investigating LEAP2 in clownfish could substantially contribute to future drug development. Therefore, this study identified and characterized a LEAP2 homolog (AoLEAP2) from clownfish, examining its antibacterial activity and immune-regulatory functions. The findings offer insights into immune response and disease management in clownfish.

## 2. Materials and Methods

### 2.1. Fish, Cells, Bacterial Strains, and Ethical Statement

This study was conducted at the Shenzhen Base of the South China Sea Fisheries Research Institute, where 190 clownfish (*A. ocellaris*) were cultured in a sterile seawater aquaculture filtration system maintained at 25 °C. The fish were fed twice daily. Fathead minnow (FHM) epithelial cells were kept in the laboratory. *V. harveyi* was isolated from diseased clownfish, while the remaining strains used in the experiment were obtained from the China Institute of Veterinary Drug Control (Beijing, China) and stored at −80 °C until use. All experimental animal protocols were strictly in accordance with the regulations of the Care and Use of Laboratory Animals of the Guangdong Ocean University, with approval from the university.

### 2.2. Cloning of the AoLEAP2 Sequence

The AoLEAP2 gene sequence was cloned from the *A. ocellaris* genome based on the published NCBI database (XP_023117300.1) and was amplified using cDNA from the spleen through PCR with the primers AoLEAP2-F/R (Table 1). Bioinformatics analysis of the LEAP2 sequence was performed as previously described [28]. 

### 2.3. RNA Isolation and Reverse-Transcription Quantitative PCR (RT-qPCR)

The tissue distribution of the AoLEAP2 gene was investigated in clownfish. Total RNA was extracted from nine distinct tissues, including brain, muscle, head kidney, intestine, gill, liver, skin, spleen, and heart tissue, collected randomly from three healthy clownfish. To examine the expression of AoLEAP2 following bacterial challenge, immune-related tissues (the gills, liver, spleen, and intestine) were isolated from three fish in the control group (injected with 0.01 mL of sterile 0.01 M PBS) and the *V*. *harveyi*-challenged group (injected with 0.01 mL of 1 × 10^6^ CFU/mL *V. harveyi*) at 6, 12, 24, 48, and 72 h post-infection (hpi). Total RNA was extracted from the samples and reverse-transcribed into cDNA for further research. The quantitative real-time PCR (qRT-PCR) analysis was performed as follows: pre-heating at 95 °C for 5 min, followed by 40 cycles at 95 °C for 5 s and 55 °C for 30 s. The primers RT-AoLEAP2-F and RT-AoLEAP2-R, designed based on the AoLEAP2 sequence, were used for the analysis. The RT-β-actin-F and RT-β-actin-R primers were utilized as an internal control to normalize the data. The relative expression of the AoLEAP2 gene was calculated using the 2^−ΔΔCt^ method.

### 2.4. Protein Modeling and Synthesis of AoLEAP2 Peptides

In accordance with other published studies on the LEAP2 protein, mature AoLEAP2 peptides (MTPLWRIMSSKPFGAYCQNNYECLTGLCRAGHCSNVHHSPSEPVKY) were synthesized at Sangon Biotech (Shanghai, China) Co., Ltd., and then divided into 2 mg samples for each tube. All the HPLC-purified peptides exhibited a purity of 98% (Supplementary Materials).

### 2.5. The Expression of Immune-Related Genes in AoLEAP2-Stimulated Clownfish

To investigate AoLEAP2’s role in clownfish immunity, individuals received an intraperitoneal injection of either 0.1 μg/mL AoLEAP2 solution or an equivalent volume of PBS. The fish were first infected with *V. harveyi* (1 × 10^6^ CFU/mL) through intraperitoneal injection, followed by treatment with either the AoLEAP2 peptide (0.1 μg/mL) or PBS (control) at 30 min post-infection. Immune tissues, including tissue from the gills, liver, and spleen, were collected from both the AoLEAP2 and PBS groups at 6, 12, 24, 48, and 72 h post-infection. Total RNA was extracted from these tissues and reverse-transcribed into cDNA for qRT-PCR analysis of IL1β and TNFα expression. All samples were collected following anesthesia with MS222.

### 2.6. Antibacterial Activity of AoLEAP2 In Vitro

To detect whether AoLEAP2 has an antibacterial function, all the bacteria in Table 2 were cultured in LB broth to mid-logarithmic phase and diluted to 1 × 10^5^ CFU/mL in PBS. All procedures were performed as described in a previous study until use [12]. The AoLEAP2 peptides underwent serial 2-fold dissolution in PBS buffer at a concentration of 100 μg/mL, as previously reported. In 96-well polypropylene microtiter plates, 100 μL of 1 × 10^5^ CFU/mL bacterial cells was mixed and cultured; then, the AoLEAP2 peptides underwent serial 2-fold dissolution, but the positive control group’s Kana^+^ concentration was maintained at 100 μg/mL. Then, the plates were cultured at 28 °C for 18 h. The lowest MIC that prevented bacterial growth was measured for the AoLEAP2 protein. The assay was performed in three replicates. 

### 2.7. Hydrolytic Effect on Bacterial gDNA

AMPs were absorbed into the cell membrane and combined with DNA, affecting DNA replication. Gel-blocking assays were used to determine whether the antimicrobial peptides had combined with the bacterial DNA. The gel shift assay was performed as previously described [10]. Briefly, bacterial genomic DNA was extracted using the Universal Genomic DNA Kit (Cwbio, Cambridge, MA, USA, CW2298M). Then, 200 ng of DNA was mixed with 50 μL of the AoLEAP2 antimicrobial peptide at different concentrations, and this mixture was then cultured at 28 °C for 1 h. Then, a 7 μL loading buffer was added, and genomic DNA mobility was analyzed using 2% agarose gel electrophoresis.

### 2.8. Cell Counting Kit-8 (CCK-8) Assay

To evaluate the cytotoxicity of the AoLEAP2 peptide, incubation was carried out using the CCK-8 Assay Kit (Beyotime, Shanghai, China). FHM cells were cultured in 96-well plates and incubated with 0.1 μg/mL, 1 μg/mL, and 10 μg/mL of the AoLEAP2 peptide for 48 h. For the CCK8 assay, CCK8 reagent was added to the plates, as previously described, and they were cultured for 4 h; then, optical density was measured using a microplate reader at a wavelength of 450 nm (Thermo Fisher Scientific, Madrid, Spain). At least five independent experiments were repeated three times, and all values are presented as the mean ± standard deviation (SD).

### 2.9. Fish Survival Assay

Thirty fish were divided into four groups for the survival assay. The fish in each group were injected intraperitoneally with 10 μL of 1 × 10^7^ CFU/mL *V. harveyi*. After 30 min, three groups received intraperitoneal injections of 10 μL of the AoLEAP2 peptide at concentrations of 0.1 μg/mL, 1 μg/mL, and 10 μg/mL, while the control group was injected with an equal volume of PBS. The fish were monitored every 24 h for 7 days to record mortality or moribundity. Survival rate analysis was performed using the Kaplan–Meier method.

### 2.10. Statistical Analysis

Statistical analyses were carried out with one-way ANOVA in SPSS 17.0. Data were presented as the mean ± standard error from three independent experiments, and statistical significance was defined as follows: *p* > 0.05—not significant; *p* < 0.05 (*)—significant; and *p* < 0.01 (**)—extremely significant.

## 3. Results

### 3.1. LEAP2 Sequence Characteristics in Clownfish

**Figure 1:**
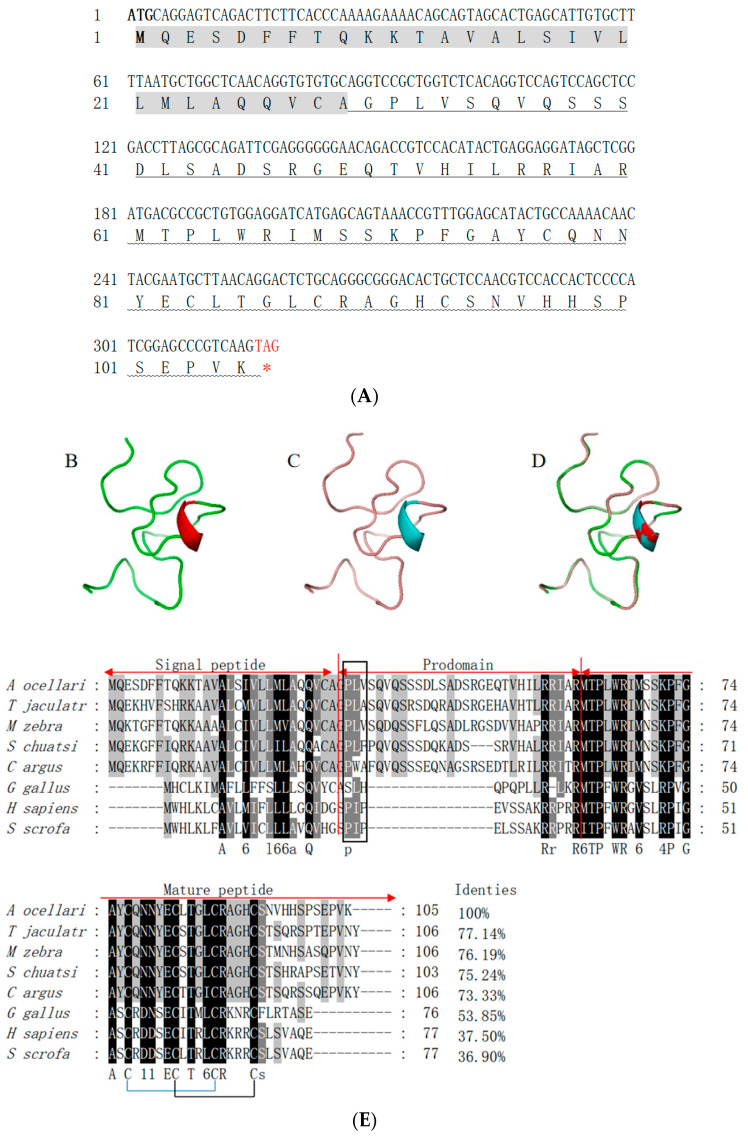
Sequence characteristics of LEAP2 in *A. ocellaris*. (**A**) Sequence analysis of AoLEAP2. The bold font represents the translation start codon. The red font represents the translation stop codon. The gray shaded part represents the signal peptide. The underlined part represents the pro-domain. The wavy line represents the mature peptide. The three-dimensional structures of the clownfish LEAP2 protein (**B**), human LEAP2 protein (**C**), and merged protein (**D**) are shown. (**E**) Multiple sequence alignment of the amino acid sequences of the LEAP2 protein among different species, with the signal peptides, pro-peptides, and mature peptides indicated above. The predicted cleavage site between them is marked with an arrowhead. Red symbol * means stop codon.

**Figure 2:**
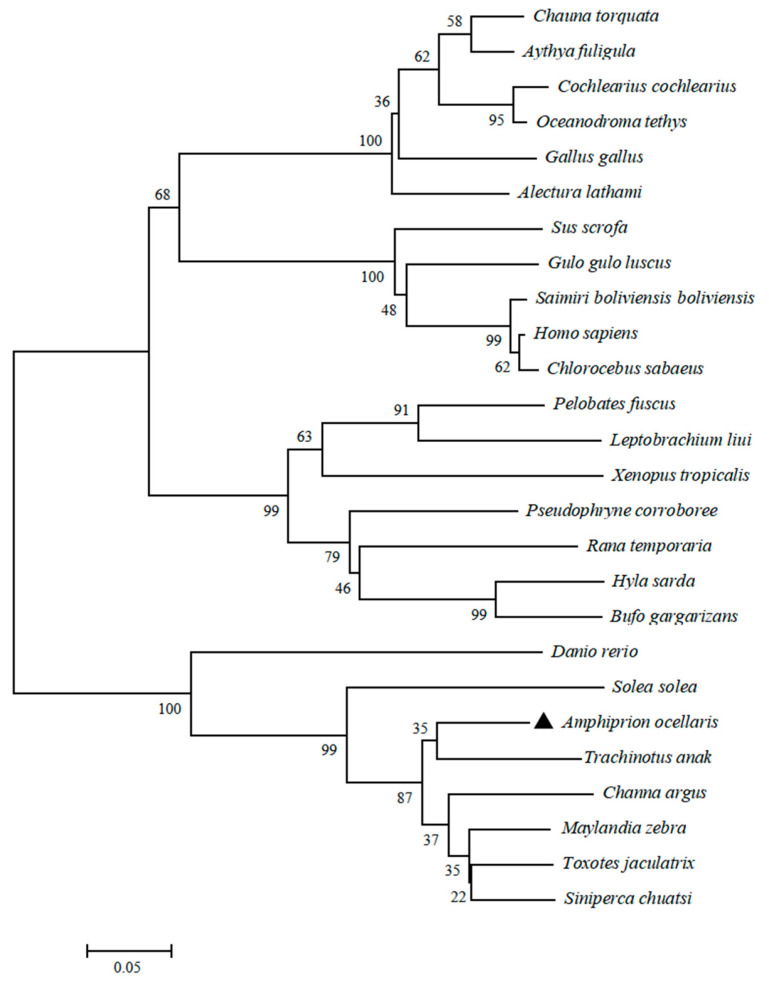
Phylogenetic tree of AoLEAP2 family members, constructed using the neighbor-joining method. Black triangle indicate subject investigated (*Amphiprion ocellaris*) in this study.

The coding region sequence of LEAP2 in *A. ocellaris* (AoLEAP2) was analyzed. At 321 bp, a 106-residue polypeptide of 11.75 kDa was encoded, with an isoelectric point of 8.39 (Figure 1A. Predicted signal (residues 1–29), pro-domain (30–60), and mature peptides (61–106) were found in AoLEAP2). The mature peptide had a putative molecular weight of 5.2 kDa and an isoelectric point of 8.21. The three-dimensional structural modeling predictions show that the AoLEAP2 (Figure 1B) protein is structurally very similar to human LEAP2 (Figure 1C), with both consisting of an α-helix, a β-turn, and an irregular coiled coil, and they also demonstrate similar standard extension results (Figure 1D). Multiple alignments with other LEAP2 amino acid sequences revealed some areas of amino acid conservation throughout the group (Figure 1E). Compared with the signal peptide and pro-peptide, the mature peptide is relatively conserved. The C-terminal mature peptide proteins, containing two characteristic cysteine residues, were involved in the formation of disulfide bonds. A comparison with the *Trachinotus anak* LEAP2 amino acid sequence showed that *A. ocellaris* was similar to *T. anak* (77.14%). A conserved RXXR motif was recognized and cleaved by furin-like endoprotease in LEAP2 to cleave the pro-peptide and produce the mature peptide. The mature peptides of AoLEAP2 contained 46 amino acid residues, and the phylogenetic tree shows that LEAP2 in *A. ocellaris* is most closely related to that in *T*. *anak* (Figure 2).

### 3.2. Expression of AoLEAP2 in Response to Bacterial Infection

**Figure 3:**
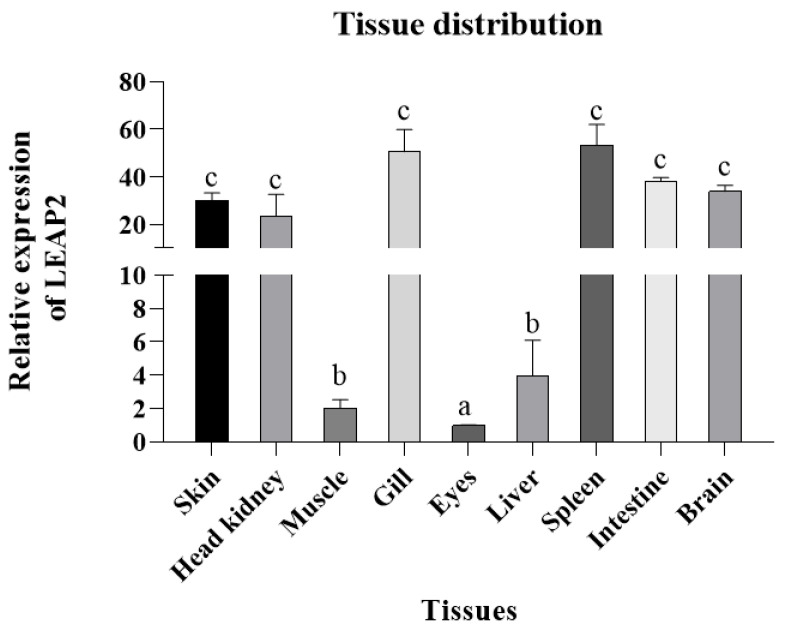
The mRNA expression analysis of AoLEAP2 in healthy fish. The AoLEAP2 gene expression in the eyes was set at 1.0. The results are expressed as the mean  ±  SEM (*n* =  3), where N is the number of times the experiment was performed. (a–c) different lowercase letters indicate significant differences between tissues (*p* < 0.05), while the same lowercase letters indicate no significant differences between tissues (*p* > 0.05).

**Figure 4:**
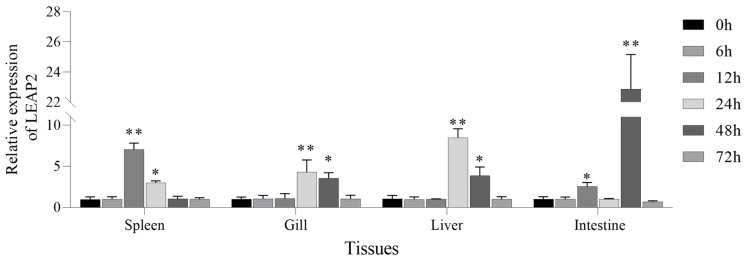
The mRNA expression analysis of AoLEAP2 in fish challenged with *V. harveyi*. Tissues were collected at different time points after bacterial infection. Data are expressed as the mean ± SEM of the results from four fish. * *p* < 0.05; ** *p* < 0.01.

LEAP2 was distributed in all tissues in *A. ocellaris*, with the highest expression observed in the spleen, followed by the gills, skin, intestine, brain, and head kidney; on the other hand, relatively low expression was detected in the muscle, liver, and eyes (Figure 3). When challenged with *V. harveyi*, *A. ocellaris* exhibited a strong transcription response, particularly in the intestine, where there was a marked increase in the expression of AoLEAP2, with maximal values of 12 hpi and 48 hpi (Figure 4), respectively. The maximum expression of AoLEAP2 was 12 hpi in the spleen, while it was even higher in the gills and liver, with values of 12 hpi and 24 hpi, respectively. The consistent and timely upregulation of AoLEAP2 in these immune organs indicates that they are integral components of the innate immune response, contributing to early defense against *V. harveyi* infection.

### 3.3. Chemically Synthesized AoLEAP2 Shows Antibacterial Activity In Vitro

An MIC assay was used to detect antimicrobial activity. It was discovered that the recombinant AoLEAP2 protein is effective against a broad range of Gram-positive and Gram-negative bacteria. Specifically, the MIC of AoLEAP2 against *Edwardsiella tarda, Escherichia coli*, *Shigella sonnei*, *Aeromonas caviae*, and *Proteus mirabilis* was 7.81 μg/mL; the MIC for *Aeromonas hydrophila* and *Pseudomonas aeruginosa* was 31.25 μg/mL; and against *Streptococcus iniae,* it was 125 μg/mL. However, the recombinant AoNk-lysin protein displayed no antibacterial effect for the rest of the tested bacteria, including *Staphylococcus aureus*, *Proteus vulgaris*, and *Salmonella typhimurium*, whose MICs were higher than 250 μg/mL (Table 2).

### 3.4. Mature AoLEAP2 Binds to Bacterial Genomic DNA

**Figure 5:**
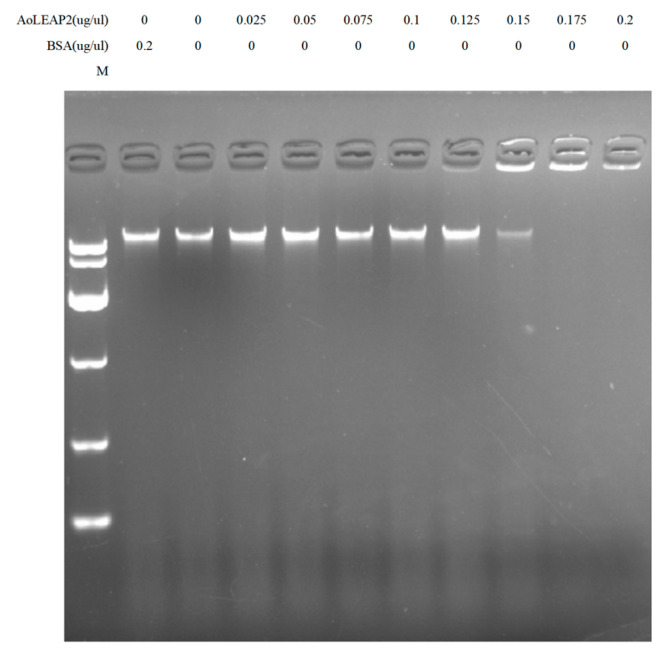
The effect of AoLEAP2 on DNA. At different concentrations, 20 μL of AoLEAP2 peptides was incubated with 200 ng of bacterial DNA at room temperature for 1 h, and the reaction mixtures underwent 2% agarose gel electrophoresis. BSA was used as a negative control.

DNA-binding assays were used to determine the mechanism of LEAP2’s antibacterial effects. It was shown that mature AoLEAP2 was strongly bound to bacterial genomic DNA (Figure 5) and that it effectively degraded 200 ng of genomic DNA at concentrations higher than 0.15 ug/uL.

### 3.5. Change in IL1β and TNFα Expression of AoLEAP2 in Fish Tissue After V. harveyi Infection

**Figure 6:**
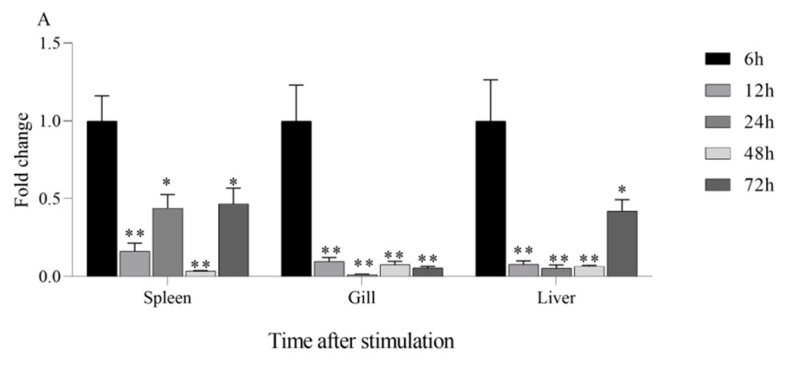

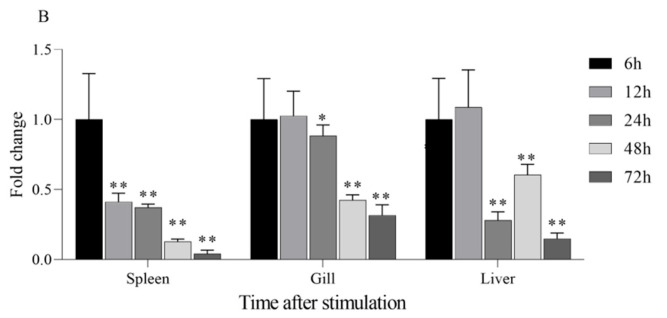
AoLEAP2’s effect on the mRNA expression of AoTNFα and AoIL1β in the liver, gills, and spleen of *V. harveyi*-infected clownfish. Each fish was first injected intraperitonially with 10 μL of live *V. harveyi* at a concentration of 1.0 × 10^6^ CFU/mL and then injected again 30 min later with an equal volume of AoLEAP2 at 0.1 μg/mL. The expression of AoTNFα (**A**) and AoIL1β (**B**) in the tissue was determined to be 6, 12, 24, 48, and 72 hpi, respectively. Data are expressed as the mean ± SEM. *n* = 3. * *p* < 0.05; ** *p* < 0.01.

AoLEAP2’s effect on the mRNA expression of pro-inflammatory cytokines such as IL1β and TNFα was investigated by qPCR. The tissue samples were collected from the fish after treatment with 10 μL of AoLEAP2 (and the same volume of PBS was injected for the control group) following infection with 10 μL of live *V. harveyi* at a concentration of 1.0 × 10^6^ CFU/mL. According to the qPCR data, the AoLEAP2 treatment significantly inhibited the *V. harveyi*-induced mRNA expression of IL1β and TNFα in diseased fish tissue compared to that in the control fish treated with PBS (Figure 6).

### 3.6. Effect of AoLEAP2 on FHM Cells

**Figure 7:**
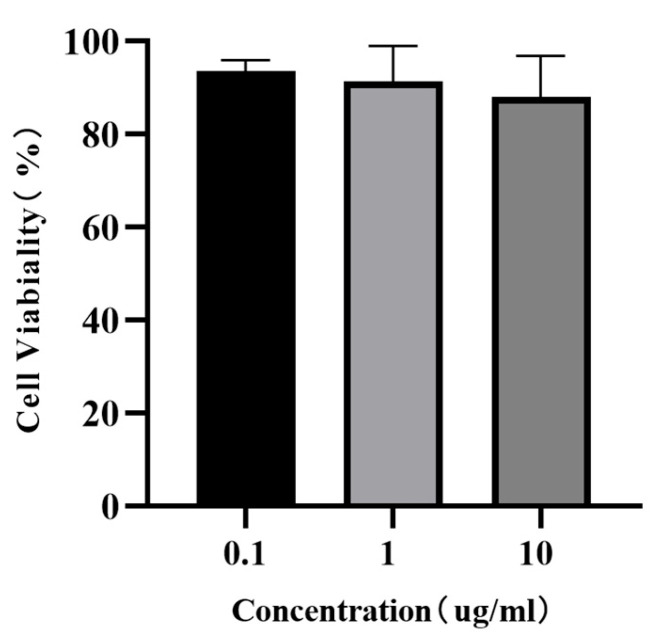
A CCK8 assay was used to test the viability of FHM cells after incubation with different concentrations of AoLEAP2. Data are expressed as mean ± SEM. *n* = 5.

Considering that antimicrobial peptides are somewhat toxic to cells, they can cause cell death. The CCK 8 assay’s results showed that all the FHM cells incubated with different concentrations of AoLEAP2 were highly viable (Figure 7), suggesting that even high concentrations of AoLEAP2 were not toxic to the FHM cells.

### 3.7. Effect of AoLEAP2 on the Survival of V. harveyi-Infected Clownfish

**Figure 8:**
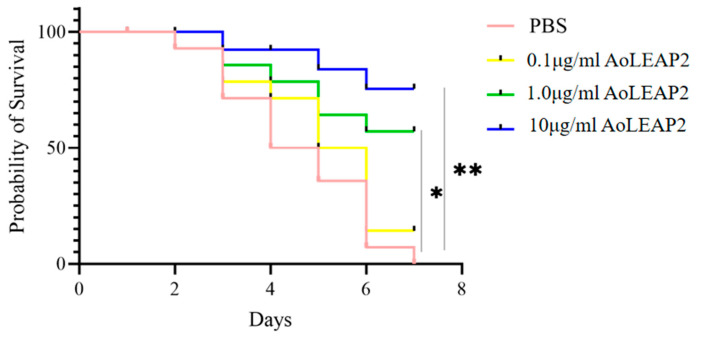
The effect of treatment with AoLEAP2 at varying concentrations on the survival rate of *V. harveyi*-infected clownfish. The fish in the experimental groups received an intraperitoneal injection with 10 μL of AoLEAP2 at concentrations of 0.1, 1.0, or 10.0 ug/mL 30 min after *V. harveyi* infection, respectively. The control group received an equal volume of PBS. The fish were monitored for signs of sickness and mortality daily for 7 days. *n* = 30. * *p* < 0.05; ** *p* < 0.01.

To investigate whether the synthesized mature AoLEAP2 peptide was effective against *V. harveyi* infection, a survival rate assay was performed. The intraperitoneal administration of the AoLEAP2 peptide at various concentrations had a significant influence on the 7-day survival rates. The infected fish behaved abnormally; for example, they exhibited abnormal swimming or skin damage, and some eventually died. All the untreated fish died by day 7. The fish treated with 10 μL of AoLEAP2 at concentrations of 1.0 and 10.0 μg/mL achieved survival rates of 57.14% and 75.52% by day 7, respectively (Figure 8).

## 4. Discussion

Bacterial outbreaks are among the most devastating diseases in aquaculture, causing significant mortality in infected fish, shrimp, and shellfish. *V. harveyi,* a severe pathogen affecting aquatic organisms, can induce life-threatening symptoms including muscle necrosis, fin rot, and liver/spleen damage in affected fish, resulting in substantial economic losses for the industry [29]. Furthermore, with the increasing consumption of aquatic products and the rapid development of aquaculture, bacterial diseases and foodborne illnesses have become increasingly severe. Currently, antibiotic use remains the primary method for treating and preventing bacterial diseases in aquaculture, yet challenges such as drug-resistant strains, superbugs, and pesticide residues have made developing alternative solutions a critical challenge in disease control. Research has revealed that antimicrobial peptides not only directly inhibit bacterial, fungal, parasitic, and viral growth but also play a vital role in innate immune responses. Recent evidence indicates these peptides can serve as effective adjuvants, synergizing with other immune factors to enhance adaptive immunity and promote wound healing. As the mechanisms of antimicrobial peptides continue to be elucidated, their potential applications in human and fish disease treatment are becoming increasingly feasible.

Antimicrobial peptides (AMPs) exhibit diverse structures, peptides, and functions, and their expression varies across tissues, with significant upregulation in immune organs following bacterial or viral stimulation. LEAP2, a well-known AMP, was initially identified in mammals and subsequently discovered in other vertebrates, including fish and frogs [5,24,30,31]. A novel LEAP2 gene has been cloned and identified in clownfish. Bioinformatic analysis revealed that AoLEAP2 contains a signal peptide, a pro-domain peptide, and a mature peptide. The structure of AoLEAP2 is similar to other reported LEAP2 sequences in the NCBI database, characterized by four conserved cysteine residues forming two pairs of disulfide bonds [24]. Interestingly, the antimicrobial activities of LEAP2 in humans are not associated with the structure of these disulfide bonds [8] but are instead dependent on its membrane affinity to bacterial cells. The phylogenetic tree analysis suggested that AoLEAP2 belonged to the fish LEAP2 group and is most closely related to *T. anak* LEAP2. The highly conserved structure and sequence of LEAP2 across species underscores its important evolutionary role in animals.

AoLEAP2 was variably expressed across all examined tissues, predominantly in immune organs such as the spleen, gills, intestine, and head kidney, similarly to other fish species [11,12,18,23]. Following *V. harveyi* infection, AoLEAP2 mRNA expression significantly increased in all tested tissues, demonstrating its potential role in combating *V. harveyi* infection. Similarly, LEAP2 expression in ayu, grass carp, and mudskipper increases significantly in the liver, kidney, and spleen upon infection by *V. anguillarum*, *A. hydrophila*, and *E. tarda*, respectively [11,12,23]. In the intestine, AoLEAP2 mRNA expression increased following *V. harveyi* challenge, consistent with findings in grass carp and common carp infected by *A. hydrophila* [11]. However, LEAP2 mRNA expression was downregulated in the intestine of ayu [12], mudskipper, and large yellow croaker [10] upon bacterial challenge. Previous studies have indicated that *V. harveyi* is a primary agent of enteritis in fish. Post-infection, the intestinal barrier is compromised, disrupting the intestinal microbiota balance and leading to high AMP expression in the intestine. These findings imply that AoLEAP2 plays a critical role in the early immune response to pathogens.

Antimicrobial peptides are a class of polypeptides known for their potent antibacterial properties and ability to modulate inflammatory responses. These peptides function by regulating key immune molecules such as cytokines and pro-inflammatory mediators. Cytokines, which are small proteins, play a crucial role in orchestrating immune responses, while pro-inflammatory mediators are compounds released during inflammatory processes. One mechanism through which antimicrobial peptides exert their anti-inflammatory effects is by inhibiting the production of pro-inflammatory cytokines, such as tumor necrosis factor alpha (TNFα), interleukin-1β (IL1β), and interleukin-6 (IL6). For example, human β-defensin 3 (hBD3) has been demonstrated to suppress the production of TNFα, IL1β, and IL6 in human monocytes. Similarly, defensins found in cows have been shown to block TNFα signaling by directly binding to its receptors. Furthermore, the frog skin peptide magainin-2 has been identified as a potent inhibitor of interleukin-1β (IL1β) and interleukin-6 (IL6) production in rat macrophages. In this study, the AoLEAP2-mediated downregulation of IL-1β and TNF-α may have contributed to host protection by preventing immunopathology, thereby further elucidating its biological function beyond direct antibacterial activity. These findings underscore the diverse mechanisms by which antimicrobial peptides can modulate inflammatory responses, highlighting their potential therapeutic applications in conditions characterized by dysregulated immune and inflammatory processes.

Previous studies have often focused on synthetic antimicrobial peptides (AMPs) derived from animals that are valued for their antibacterial properties [32]. Similarly, mature LEAP2 peptides from aquatic animals have demonstrated effective In Vitro antibacterial activity. Our study found that AoLEAP2 exhibits broad-spectrum antibacterial activity against both Gram-positive and Gram-negative bacteria, with a lower effective dose required for Gram-negative bacteria, potentially due to the protective cytoderm in Gram-positive bacteria. Prior research has suggested that AMPs disrupt bacterial cell membrane integrity [31], although some reports indicate that they also inhibit DNA replication by binding to DNA [31]. Our findings show that AoLEAP2 effectively binds to genomic DNA, inhibiting its migration, suggesting that LEAP2 plays a crucial role in inhibiting DNA replication. While our gel retardation assays clearly demonstrate that AoLEAP2 binds to bacterial genomic DNA, potentially interfering with DNA replication and transcription, we acknowledge that membrane disruption represents another important antimicrobial mechanism employed by many AMPs, including LEAP2 family members in other species. Although we did not directly assess membrane permeability in this study, the structural features of AoLEAP2 (e.g., its cationic and amphipathic properties) suggest that it may also possess membrane-interacting capabilities. This dual potential for both DNA-binding and membrane-targeting mechanisms is consistent with the multifunctional nature of many antimicrobial peptides. Future studies employing membrane integrity assays (e.g., propidium iodide uptake or SYTOX green staining) could help elucidate whether membrane disruption contributes to AoLEAP2’s antimicrobial activity. It is well established that the immune system can be activated by increasing the expression of pro-inflammatory cytokines, such as IFN-gamma, IL22, IL8, IL1β, and TNFα, as well as Th2 cytokines [33]. The mRNA expression levels of interleukin 1 beta (IL1β) and tumor necrosis factor alpha (TNFα) were chosen as indicators of systemic immune activation in the fish. The results from this study demonstrated that high doses of AoLEAP2 significantly enhanced survival rates following bacterial challenge with *V. harveyi*. Moreover, the mRNA expression levels of AoIL1β and AoTNFα were notably decreased in various tissues from the infected fish. These results collectively suggest that LEAP2 not only facilitates bacterial clearance but also reduces the expression of inflammatory mediators, thereby contributing to whole-body protection. Furthermore, the cytotoxicity of AoLEAP2 in FHM cells was evaluated using the CCK8 assay, revealing that the viability of FHM cells across all experimental groups remained above 80% after exposure to varying concentrations of AoLEAP2. This indicates that AoLEAP2 can be safely administered to fish.

## 5. Conclusions

In summary, a novel liver-expressed antimicrobial peptide 2 (AoLEAP2) was cloned and characterized in clownfish, and it was found to be highly expressed in the gills and spleen. In addition, the expression of AoLEAP2 was significantly upregulated in immune organs after *V. harveyi* infection. The chemically synthesized AoLEAP2 had broad-spectrum antimicrobial activity and was able to bind to DNA effectively. AoLEAP2 treatment significantly inhibited the *V. harveyi*-induced mRNA expression of IL1β and TNFα in diseased fish tissues compared to their expression in the control fish treated with PBS. Furthermore, not only does AoLEAP2 exhibit antibacterial properties but it also plays a critical role in immune regulation by mitigating hyperinflammatory responses, enhancing its therapeutic potential for bacterial infections. These data will provide a reference for the control and prevention of fish diseases.

## Figures and Tables

**Table 1:** Primers used for AoLEAP2 gene cloning and qRT-PCR analysis.

Primer Name	Sequence (5′-3′)	Tm (°C)	Products (bp)	Used
*AoLEAP2-OF*	ATGGAAAGAATTTCAATCCTG	56	324	Amplification of ORF of AoLEAP2
*AoLEAP2-OR*	TCCATGCATAGGATGTACAG			
*RT-AoLEAP2-F*	GGGAACAGACCGTCCACATAC	55	135	Real-time PCR
*RT-AoLEAP2-R*	GCAGAGTCCTGTTAAGCATTCG			
*RT-AoIL1β-F*	CAGTGACAACCGCAAAGT	53	140	
*RT-AoIL1β-R*	GAGATTAGTGTCCCTGATGC			
*RT-AoTNFα-F*	GCCCAACAGGAACG	54	120	
*RT-AoTNFα-R*	TTCACGCAGATTACGAT			
*RT-β-actin-F*	GGGCCAAAAGGACAGCTAC	57	156	
*RT-β-actin-R*	CAGGGTCAGGATACCCCTCT			

**Table 2:** The minimal inhibitory concentration of the AoLEAP2 protein against a panel of microorganisms.

Bacterial Strains		Minimal Inhibitory Concentration (MIC; μg/mL)	
		AoLEAP2	Kana^+^
Gram-positive bacteria	*Staphylococcus aureus* ATCC6538	>250	+
	*Streptococcus iniae* ATCC29178	125	+
Gram-negative bacteria	*Edwardsiella tarda* ATCC15947	7.81	+
	*Escherichia coli* ATCC8739	7.81	+
	*Aeromonas hydrophila* ATCC8739	31.25	+
	*Shigella sonnei* CMCC(B)51529	7.81	+
	*Pseudomonas aeruginosa* ATCC27853	31.25	+
	*Aeromonas caviae* BNCC139095	7.81	+
	*Proteus vulgaris* CMCC(B)49027	>250	+
	*Salmonella typhimurium* ATCC14028	>250	+
	*Proteus mirabilis* CMCC(B)49005	7.81	+

## Data Availability

The data that support the findings of this study are available on request from the corresponding author. The data are not publicly available due to privacy or ethical restrictions.

## Supplementary Materials

The following supporting information can be downloaded at: https://www.mdpi.com/article/10.3390/ani15172590/s1, Sample Information.
