# Supplementary material for: Molecular Characterization of the Liver-Expressed Antimicrobial Peptide 2 (LEAP2) from Amphiprion ocellaris and Its Role in Antibacterial Immunity
Source: Animals (Basel). 2025 Sep 3;15(17):2590. doi: 10.3390/ani15172590 (PMC12427373; doi:10.3390/ani15172590)
Supplement: Supplementary file 1 [file animals-15-02590-s001.zip › animals-3843145-supplementary.pdf]

HPLC-AoLEAP2

Sample Information

Name	:	P30088
Sequence	:	MTPLWRIMSSKPFGAYCQNNYECLTGLCRAGHCSNVHHSPSEPVKY
Modification	:	N/A
Lot.No	:	P30088-23081701
Pump A	:	0.1%trifluoroacetic in 100%water
Pump B	:	0.1%trifluoroacetic in 100%acetonrtrile
Total Flow	:	1.0ml/min
Wavelength	:	214nm
Analytical column type	:	NanoChrom Chromcore TM120 C18(4.6*250MM*5UM)
Dissolution method	:	0.1mg sample dissolved to 0.5mL by 10%ACN and 90%H2O
Acquisition Time	:	2023/09/07 14:17:58
Inj.Volume	:	30ul

Time	Module	Action	Value
0.01	Pumps	B.Conc	30
20.00	Pumps	B.Conc	50

Chromatogram

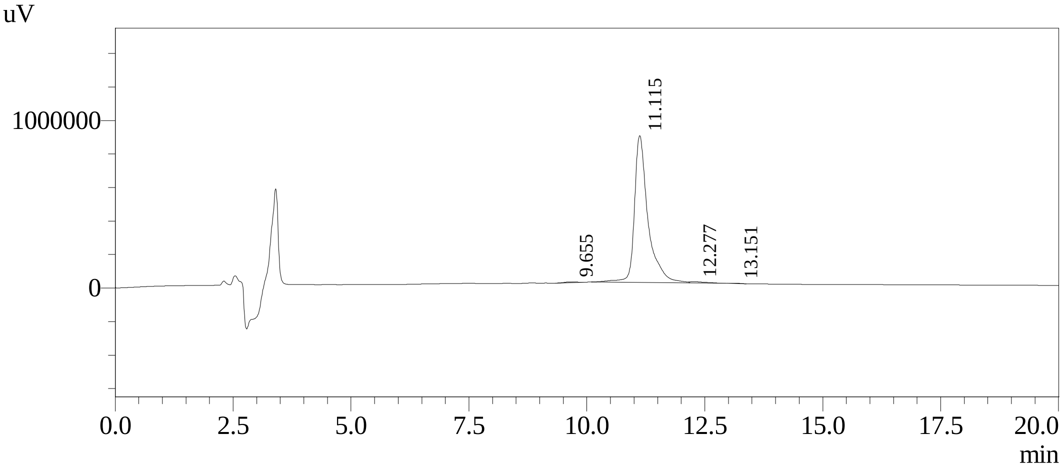

1 Det.A Ch1 / 214nm

PeakTable

Detector A Ch1 214nm

Peak#	Ret. Time	Area	Height	Area %	Height %
1	9.655	80165	4591	0.449	0.515
2	11.115	17566969	876203	98.374	98.226
3	12.277	186725	8774	1.046	0.984
4	13.151	23522	2457	0.132	0.275
Total		17857381	892025	100.000	100.000

LCMS-AoLEAP2

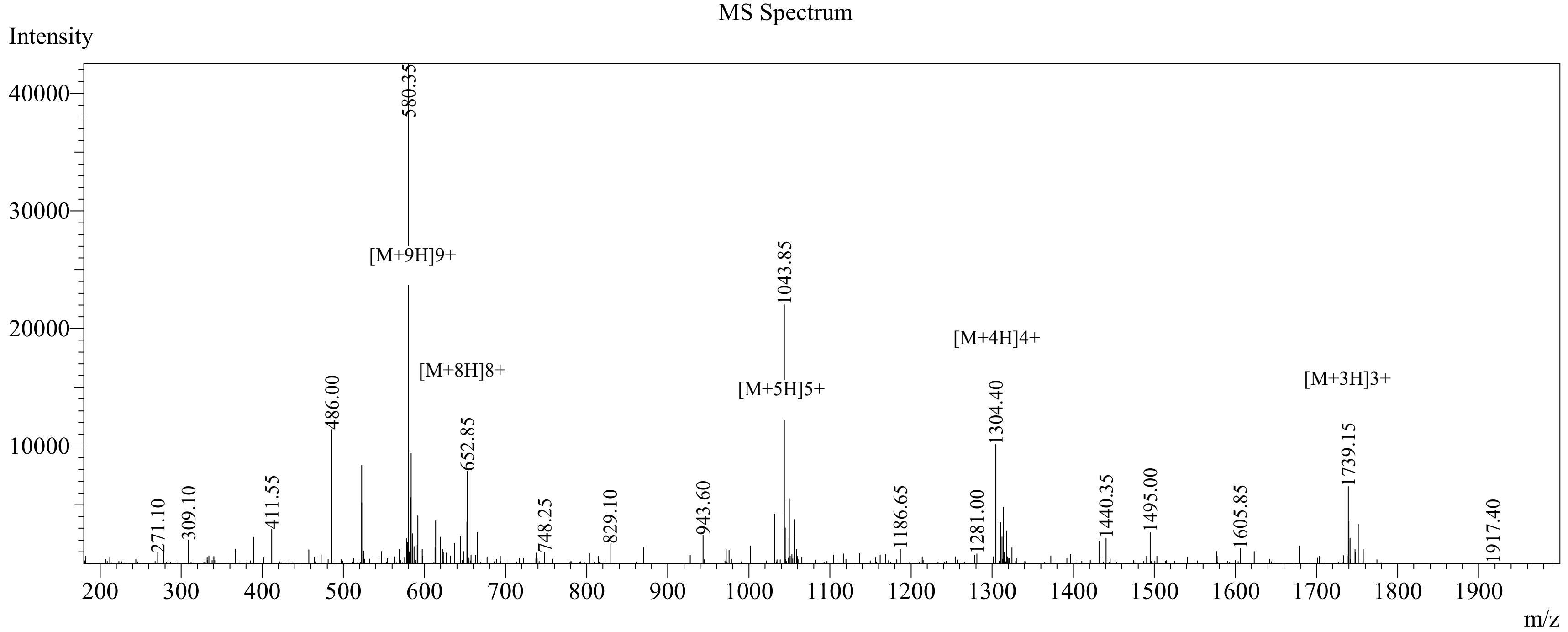

Sample Information
Dissolution method :0.1mg sample dissolved to 0.5mL by 50%ACN and 50%H2O
Date Acquired :2023/09/07 14:54:09
Injection Volume :1ul

Interface :ESI
Nebulizing Gas Flow :1.50L/min
CDL Temp :250℃
CDL Volt :0v
Block Temp :200

Prerod Bias :+4.5kv
Detector :-0.2kv
T.Flow :0.2ml/min
B.conc :50%H2O/50%MeOH

Name :P30088
Sequence :MTPLWRIMSSKPFGAYCQNNYECLTGLCRAGHCSNVHHSPSEPVKY
Modification :N/A
Lot No. :P30088-23081701
Theoretical :5214.998
bserved :5214.15
